# A health promotion intervention to address youth violence among students in a technical college in Sri Lanka guided by the participatory action research approach: a study protocol

**DOI:** 10.1186/s40900-022-00393-3

**Published:** 2022-10-22

**Authors:** Nadeeka Rathnayake, Kalpani Abhayasinghe, Jayamal De Silva, G. N. Duminda Guruge

**Affiliations:** 1 Department of Health Promotion, Faculty of Applied Sciences, Rajarata University of Sri Lanka, Mihintale, Sri Lanka; 2 Department of Nursing & Midwifery, Faculty of Allied Health Sciences, General Sir John Kotelawala Defence University, Ratmalana, Sri Lanka; 3 Institute for Research and Development in Health and Social Care, Battaramulla, Sri Lanka; 4 Department of Psychiatry, Faculty of Medical Sciences, University of Sri Jayewardenepura, Colombo, Sri Lanka

**Keywords:** Action research, Empowerment, Health promotion, Qualitative, Students, Technical colleges, Youth violence

## Abstract

**Background:**

Youth violence is a global public health issue and the highest rates are reported in Low and Middle-Income Countries (LMICs). Higher rates of youth violence are reported in Sri Lanka as well. Students who fail to continue higher studies in schools or enter the universities in Sri Lanka, enroll in technical colleges and are associated with a higher number of risk factors of violence. This study aims to empower youth (15–29 years old) of a technical college in Matale district, Sri Lanka, to carry out activities among themselves to improve their knowledge, change perceptions, and violence-related behaviours.

**Methods:**

The Participatory Action Research (PAR) approach will be used. The study participants will be eighty students in a technical college in Matale district, Sri Lanka. The study period will be three years. Study participants will also be collaborators and they will involve actively in all stages of the study. A health promotion intervention will be implemented to identify determinants of youth violence and to design and implement actions while monitoring the changes. The data will be collected mainly through focus group discussions and key informant interviews both before and after the health promotion intervention. Additionally, a self-administered questionnaire will be used and the principal investigator will maintain a reflective diary. The qualitative data will be analysed thematically whereas quantitative data will be analysed using descriptive statistics. Data will be triangulated to increase the rigour of the study.

**Discussion:**

According to literature, PAR is not widely used in health promotion. The enabling and empowerment goals of health promotion are fulfilled in PAR. Thus, this will be a novel experience for researchers and this will stimulate discussion on the combination of PAR and health promotion. This study design itself promotes active participant involvement and it may generate effective youth-led, culturally appropriate actions to address youth violence. The findings will describe what works and why it works and will help Sri Lanka and similar LMICs to create safe environments for youth in educational institutes or training colleges.

## Plain English Summary

In this protocol paper, we propose a participatory action research based on the health promotion approach to address youth violence in Sri Lanka which has hitherto not been addressed effectively. We selected this topic in the context where youth violence has become a major public health issue in low and middle-income countries. Since, empowering young people in preventing violence is vital, this study aims to empower the youth of a technical college in Matale district, Sri Lanka to carry out activities among themselves to address youth violence. Thus, we will select eighty students from a Sri Lankan technical college for the study. The students will involve themselves in all stages of the study as research collaborators. We will collectively identify the risk factors of youth violence, design and implement actions to address those risk factors with active youth involvement. The data will be mainly collected before and after the actions to identify the changes in knowledge, perceptions and behaviours that have been taken following the actions. The students who participate in the study will ultimately gain knowledge and skills to address violence in their college and other settings. Further, it will reduce their risk of becoming an offender or a victim of violence. This study is expected to generate effective youth-led, culturally appropriate actions to address youth violence in a selected setting. The findings will be helpful for Sri Lanka and similar low and middle-income countries to create safe environments for youth in educational institutes.

## Background

The world report on violence and health defined youth violence as “violence that occurs among individuals aged 10–29 years who are unrelated and who may or may not know each other and generally takes place outside of the home” [1]. There is no internationally agreed definition for the youth age group. The United Nations defined youth as persons in the age range of 15–24 years [2]. The National Youth Policy of Sri Lanka defined people in the age range of 15–29 years as the youth considering the transition from a dependent child to an independent adult in the Sri Lankan context [3]. Sri Lanka has 4.64 million young people in the age range of 15–29 years accounting for 23.2% of the total population [4]. Thus, those between the age range of 15–29 years will be considered as youth in the present study.

Youth violence is one amongst the foremost visible types of violence in society. Media report daily on violence in schools, violence by gangs or youth on the streets. The victims and perpetrators of such violence are adolescents or young adults [5]. Youth violence is a global public health issue including a range of violent acts from school bullying and fighting to more severe sexual and physical assault with or without a weapon, dating violence, gang violence and homicides [1, 6, 7]. Sexual violence also influences a major proportion of youth [6].

Worldwide, youth violence causes an estimated 200,000 homicides among youth in the age group 10–29 each year and a majority of these deaths occur in Low and Middle-Income Countries (LMICs) [6, 8]. Apart from the United States, most of the countries which report youth homicide rates above 10.0 per 100 000 are either developing countries or countries which undergo rapid socio-economic changes [1]. When Asian countries are considered, the reported prevalence of physical fighting among youth [13–16 years old] is as follows; China 16.59%, Thailand 33.30%, Indonesia 33.76%, Sri Lanka 47.28% and Philippines 50.02% [9], where the second highest prevalence out of the countries surveyed has been reported as Sri Lanka.

Violence seems to be growing among Sri Lankan youth [10, 11]. In Sri Lanka, according to the Global School-based Student Health Survey (GSHS) 2016, which was conducted in government schools in 17 districts with students of grades 8 to 12 (n = 3125), the percentages of students (13–17 years old) who were physically attacked and in a physical fight one or more times during the past 12 months before the survey were 35% and 43.8% respectively [12]. The National Youth Health Survey in Sri Lanka,2013 has revealed that 3.7% of male and 1.5% of female young people aged 15–19 years had been involved in a fight during the past 12 months which needed medical treatment [13]. A study done on violence among 13–25 years old youth in the district of Gampaha, Sri Lanka has shown the prevalence of victimization to any act of violence at least once within the last six months as 85.1% [14]. A study done with 630 adolescents (mean age: 16.5 years) in the district of Kalutara, Sri Lanka has found that more than 50% of the adolescents had been victimized or perpetrated physical violence during the last six months [15]. Further, ragging is a form of violence among university students in Sri Lanka. In a study done using 623 students at the Jaffna University of Sri Lanka, it has been found that 59% of the students have experienced emotional or verbal ragging [16]. Another study done using 1322 Sri Lankan undergraduates of a mean age of 21.8 years has found that 44% and 36% of participants had experienced sexual and physical harassment respectively [17].

Individual characteristics such as having a history of aggression and beliefs that support violence lead to youth violence [18]. The long-term predictors of youth violence include low intelligence, higher impulsiveness and biological factors like low heart rate [19]. Having faced traumatic events and mental illnesses such as depression also lead to youth violence. [6, 20, 21]. The family factors that contribute to youth violence include harsh parenting practices, insufficient parental supervision, parents’ use of alcohol, domestic violence, divorce or other family disruption, young mothers and larger families [18–21]. Victims of child maltreatment are more likely to perpetrate youth violence when compared to non-victims [18, 22]. School bullying is a correlate of youth violence [23]. Peer pressure, having delinquent peers and problems at school like chronic discipline issues are the risk factors of youth violence [18–20]. Poverty, urban residence and neighbourhoods with higher crimes also cause youth violence [19] and youth violence can have its roots in cultural characteristics and societal conditions like poverty, discrimination or both [24]. Youth violence not only contributes hugely to the global burden of premature deaths and disability but also causes long-term impacts on a person’s psychological and social functioning. It negatively affects whole communities by increasing the costs of health and justice services and reducing productivity [6, 7, 25].

In social institutions where relationships are formed, violence is an integral part [26]. Low educational achievement, low commitment to school and school failure are also risk factors for youth violence [6]. The Sri Lankan technical colleges to which the students who generally fail to continue their higher studies in schools enroll [27] to receive vocational training [28] may even be more potentially risky settings of violence than schools.

Vocational training is provided in some LMICs for disadvantaged youth to equip them with skills to find employment opportunities and to reduce youth unemployment and poverty [8]. Joblessness is associated with youth violence and having secure employment is a protective factor [8]. As per the views of the vocational training followers in Sri Lanka, the training received has not been useful for a majority of the trainees to find employment or a livelihood opportunity [27]. Unemployment, low family income and income inequality also have been identified as risk factors for youth violence [6]. It is widely articulated in Sri Lanka that the injustice felt by disadvantaged youth leads to intolerance and violence [29]. Youth violence is largely influenced by the disadvantageousness of youth [30]. In a way, students in Sri Lankan technical colleges are a disadvantaged youth group and they have been associated with a greater number of risk factors of youth violence such as joblessness [8, 27], low family income [6] and low educational achievements [6, 27]than school or university students. Exposure to multiple risk factors increases the chances to become violent [31]. Although the empirical evidence on the prevalence of youth violence in Sri Lankan technical colleges is unavailable, technical colleges that lie between schools and universities can be considered potential sites of youth violence.

Though the highest rates of violence and highest numbers of youth are in LMICs, more research on youth violence has been conducted in High-Income Countries (HICs) [32, 33] and only a limited number of studies have been done on preventing violence in LMICs [34]. Given the huge burden on victims, communities, economy and health systems in LMICs due to youth violence, this area should be considered a priority area in health and social research [33]. The present study will contribute to filling that gap in the literature as well.

It is still questionable as to why youth choose violence to solve their problems. Additionally, the steps that need to be taken to address violence is also a question [10]. The promising individual-level interventions to address youth violence are behavioural skill training and employment programs [35]. Parenting programs, school-based bullying prevention programs, therapeutic approaches and community interventions also have been identified as promising strategies to prevent youth violence [8]. Community interventions to prevent youth violence need to integrate all primary institutions like families, schools, health care agencies, workplaces and judicial services. Examples of such programs are family support programs, community development programs and school clinics [36]. The ecological model for understanding violence describes youth violence as a result of a complex interplay of individual, relationship, community and societal factors [1, 37]. Thus, youth violence prevention needs a comprehensive approach that addresses the social determinants of violence [6]. In health promotion, interventions are built up based on the determinants or causes of health [38]. Using the empowerment model of health promotion is effective in addressing complex problems and making sustainable changes [39].

The Participatory Action Research (PAR) approach involves participants actively in the research process [40]. It best suits studies aiming to address practical problems of communities [41] and it promotes the power and voice of marginalized youth groups as well [42, 43]. An extensive review of the literature has found only a few instances of evidence that PAR has been used in health promotion programs over the years [41].

## Theoretical foundation

The principles and models that establish the link between PAR and health promotion are discussed in this section. Kurt Lewin created the term ‘action research’ [44] and defined action research as, “comparative research on the conditions and effects of various forms of social action, and research leading to social action” [45]. During the last thirty years, the interest being paid on PAR and its application has considerably grown [40].

### How does PAR differ from other research approaches?

PAR aims to resolve problems rather than just investigate them, that is how it differs from other research designs [41]. Traditional power division in research relationship is challenged in PAR [46] and participants are considered collaborators in the research [47]. PAR makes participants involve actively in the research process at various levels which might include topic selection, reflection, data collection, analysis and deciding actions whereas, in most other health research, participants are involved as passive respondents or subjects [40, 48]. In the present study, participants will be the research collaborators and they will contribute to all stages of research from the beginning to the end.

### Combining PAR with health promotion

The goal of the PAR is to provide workable solutions to concerns and to develop the capacities of people. Community-based action research strategies are driven by the priorities of the communities rather than by outside agencies or experts [41]. PAR aims at understanding and improving the world by making changes. It is a process of empowering people to have increased control over their lives [49, 50].

The Ottawa Charter in 1986 defined health promotion as, “the process of enabling people to increase control over, and to improve, their health” [51]. Health promotion is a community-based model of enhancing participation and empowering lay communities to address health issues [52]. Action research fits perfectly with health promotion [41] because the empowering and enabling goals of health promotion are fulfilled in PAR [53]. Further, in contrast to the top-down approaches, health promotion is a bottom-up approach [54] and the characteristics of PAR also strongly support it [48].

Community empowerment is at the core of health promotion and it goes beyond the health sector responses, making it a collective responsibility where communities also need to contribute actively [38, 51]. Health promotion approach makes communities involved in deciding priorities, taking decisions, planning and implementing actions [51]. In health promotion, determinants of health are focused on when interventions are developed [38]. Therefore, enabling people to increase control over the determinants of health is the basis of health promotion [51]. Taking these principles and the health promotion action areas [51] into account, Samarasinghe and colleagues, 2011 [52] introduced a community-centred model of health promotion. This model includes the basic steps; 1. Engaging with the community, generating community enthusiasm and developing collective goals with community groups to improve their wellbeing, 2. Identifying and prioritizing the underlying determinants of health issues to be addressed, 3. Developing indicators mutually with communities to identify changes in selected determinants and their wellbeing and 4. Planning and implementing community-based interventions to address selected determinants of wellbeing while monitoring the process [52]. In the model [52], determinants of health are focused on from the outset. Throughout, all these steps, the community plays an active role and it facilitates the community taking control over the whole process [52, 55]. This model will be adapted for the present study.

These steps of the community-centred health promotion model [52] have similarities with the PAR stages. For example, Lewin in 1946 [44], proposed an iterative spiral process in conducting action research including three basic steps: planning, acting, and evaluating [56]. Elliot in 1991[36], presented another model for action research sharing the features of Lewin’s work [44]. It includes, “identifying a general idea, reconnaissance or fact-finding, planning, action, evaluation, amending the plan and taking second action step, and so on” [36]. This model also shares common features with the steps of the community-centred health promotion model [52, 55].

The action researchers can adopt or adapt the models that best suit the study purpose [57]. Both spiral PAR stages and steps of the community-centred health promotion model [52] which go up and down based on effectiveness make the process very flexible. Community-centred health promotion model [52] has been adopted in several studies to address different health problems such as low birth weight [55, 58], child neglect [59, 60] and alcohol consumption [61] in Sri Lanka but not along with the PAR approach. Youth violence is a global public health issue [6] and it is a problem in Sri Lanka as well [12]. Even though health promotion interventions have been implemented in Sri Lankan schools to address bullying [62], the PAR approach has not been incorporated with those.

The dissemination of this study protocol is intended to generate interest among researchers, activists, scientists or the general community who engage in health promotion programs to use the PAR approach and to stimulate discussion on the combination of health promotion programs with the PAR approach to address complex health issues such as youth violence. The paper first outlines the research objectives. Then, proceeds to explain the research approach, study setting, sample, steps of the health promotion intervention, developing data collection tools, implementation fidelity, methods of data collection and analysis along with the study timeline. Finally, concludes with strengths, limitations and challenges encountered with suggested solutions.

## Methods

PAR involves participants in research from the onset of the study from topic selection [48]. In the present study, the principal investigator will reach participants with a broader research topic, ‘youth violence’. However, the participants can choose which aspects/types of youth violence are to be addressed in their college. The specific objectives given below might alter as per the agreement of the study participants when the study is started in the setting.

### Research question

How can youth at technical colleges in Sri Lanka be empowered to carry out and sustain a health promotion intervention to improve knowledge, and change perceptions and behaviours that cause youth violence?

### General objective

To empower youth [15–29 years old] of a technical college in Matale district, Sri Lanka to improve knowledge, and change perceptions and behaviours associated with youth violence by using PAR to implement and evaluate a health promotion intervention.

### Specific objectives

- To describe the existing knowledge, perceptions and behaviours associated with youth violence among students and identified key informants in the technical college.
- To identify the determinants of youth violence with students in the technical college.
- To enable students to design and implement actions to address selected determinants of youth violence.
- To describe the changes in knowledge, perceptions, behaviours and addressed determinants of violence following the intervention among students in the technical college.

### Research approach and study design

The PAR approach will be the research approach for the present study. PAR is defined as “a participatory, democratic process concerned with developing practical knowing in the pursuit of worthwhile human purposes” [47].

PAR encourages the use of both qualitative and quantitative approaches and advocates the use of a range of methods to collect and analyse data [41]. However, the emancipatory and participatory nature of the PAR lends itself more favourably to the qualitative approach. When using PAR in health promotion, flexible methods of incorporating both qualitative and quantitative methods of data collection have been recognized as an appropriate way [63]. Thus, mixed methods will be the study design for the present study.

### Study setting and population

Sri Lankan technical education has a history of more than 125 years and commenced with the establishment of the “Technical School” at Maradana in 1893. Currently, there are 39 technical colleges in Sri Lanka managed by the Department of Technical Education and Training which functions under the State Ministry of Skills Development, Vocational Education, Research and Innovation. To enroll for courses in technical colleges, the students need to be educated up to a minimum of grade 9 or 10 in school [28].

Actions have been taken to modernize the courses in technical colleges as suitable for industrial development. Thus, technical colleges offer many job-targeted courses in the fields of Information and Communication Technology, Plumbing, Engineering, Quantity Surveying, Landscaping, Automobile Repair and Maintenance, etc. for students who fail to continue their studies in schools or state universities [28].

A technical college in the Matale district, Sri Lanka will be selected as the current study setting based on the availability of the two-year-long courses and student counts. The total student count is varied from 400–500 in the technical colleges in the district. The study population will be the students [15–29 years old] who will retain for at least two years (who follow Diplomas or Higher Diplomas) in the college from the onset of the study.

### Sampling and sample size

All students who follow two-year courses in the college will be considered eligible to take part in the study. There will be no specific exclusion criteria other than not providing consent to participate in the study. The participant recruitment will be done by the principal investigator after obtaining permission from the relevant authorities. The principal investigator will openly invite the eligible students by visiting their classes, provide them with information about the study and recruit them with their written consent. The academic staff of the college will not be involved in the recruitment process and it will be a voluntary decision by the participants. The approximate sample size will be 80 students [50 female and 30 male students] as per the information obtained from relevant authorities.

### Steps of the health promotion intervention for the present study

**Fig. 1:**
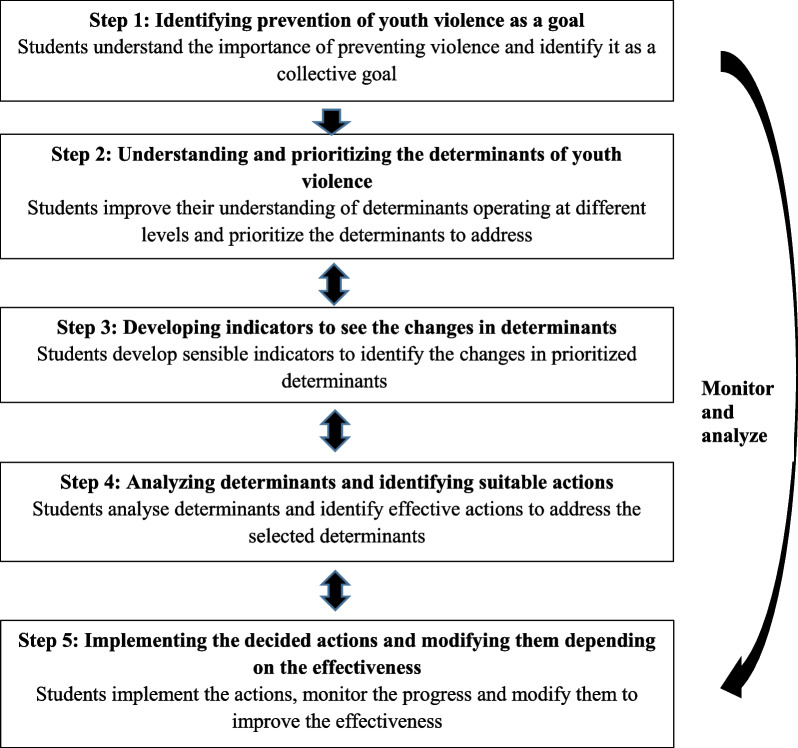
Logical framework of the health promotion intervention

The community-centred health promotion model introduced by Samarasinghe and colleagues in 2011 [52] which has been used in Sri Lankan context with success [55, 58–60] will be adopted for the present study. Figure 1 shows the logical framework of the health promotion intervention for the present study.

What will be done at each step of the logical framework is explained in Table 1.

**Table 1:** Actions and possible outcomes at each step of the logical framework

Steps	Actions and possible outcomes
Step 1	The students might not have “violence” on their agenda as a priority to be addressed. What we would do here is to take initial steps to approach the student group and then engage them in a program to improve their health and wellbeing to initiate this process in a positive aspect focusing on available resources [64]. Then, we would proceed to introduce the topic of violence and continue engagement with the group facilitating them to take over the ownership. In this step, 04 interactive discussions generally on the topics of, health and healthy youth, features of a healthy college, types of youth violence and harm of youth violence will be facilitated. Thereafter, youth violence will be identified as a matter of concern and a collective goal to have ‘a college that discourages violence’ will be set with students. Even, the most common types of violence in the college will be identified with students.
Step 2	Before the implementation of the main components of the intervention, determinants of youth violence will be identified. Group discussions will be used as the main method to identify determinants. In group discussions, one group might have a maximum of ten members and students will be allowed to make groups as convenient for them. Discussions will be conducted in classrooms or outdoor places within the college premises. Short stories (stories that describe the incidents of youth violence prepared with students) could be used to facilitate group discussions. Determinants operating at different levels like individual, family, college and society will be clarified. The gender, other social stratifiers and underlying power structures [65] which may increase the risk of becoming an offender or victim of youth violence will also be discussed. College and societal level determinants that can be addressed by youth themselves to make a change in the culture associated with youth violence could be prioritized based on the importance and changeability. For example, possible determinants to prioritize are: Social norms related to violence [1, 66] Attractive image associated with violence [20, 67] Privileges associated with violence [68] Peer influences [6, 20] However, these determinants might change in the discussions with students. The number of sessions will vary from 3–4.
Step 3	In group discussions, the indicators to identify the changes in prioritized determinants will be set. The possible indicators are: Openly criticizing violence (not justifying any form of violence) No value-added to any violence De-glorification of violence Understanding violence promotions in media No privilege or specialty is given to violent students Changes in reactions to violent acts Identifying violence as a foolish means to deal with problems and only weaker ones use it Ability to cope positively with acts of violence Good relationships among students Incorporating addressing/demoting youth violence into the agendas of teachers Changes in college rules such as changes in harsh punishments to violent students into healthier means Capability of students themselves to change behaviours of violent peers Approximately there will be 3 sessions.
Steps 4 and 5	Steps 4 and 5 will be conducted simultaneously. First, students will analyse the prioritized determinants and will identify possible actions to address those. Then, they will implement the actions while monitoring the changes. If satisfactory changes are not identified, the actions could be modified or new actions could be planned. Based on the reflections, students may go up and down in the logical framework (they can even go back to step 02 to understand and prioritize the determinants again). To monitor the changes, the indicators developed in step 03 will be used. The number of discussions will be around 10. In the discussions, actions will be designed, implemented actions will be reflected, changes will be identified and the principal investigator will also provide inputs.
Ensuring the sustainability	To make the process sustainable, it is important to involve the academic staff and the administration as well. Thus, two discussions will be facilitated with the academic staff and the administration about the possible actions at their levels to reduce violence within the college in between the discussions with students. During those discussions, their suggestions will also be taken on implemented activities. Further, at the mid of the intervention period and the end, another two discussions will be facilitated with students on how to spread the interventions to others (especially to their peers in and out of the college). They will be capacitated with sufficient knowledge and skill to address violence in their everyday settings in those two discussions.

### Developing data collection tools

The purpose of PAR is to enable action. It is implemented in a reflective cycle where participants collect data, analyse data and determine the actions to follow [48]. Thus, in the present study, all participants will involve in developing data collection tools. Thereafter, the principal investigator will select a student research group of ten members based on their enthusiasm, interest and understanding of the topic. The student research group will involve in the other steps of the research such as data collection and data analysis with the principal investigator.

In the present study, Focus Group Discussions (FGDs) and in-depth, semi-structured Key Informant Interviews (KIIs) will be used as the main data collection methods to collect data about knowledge, perceptions and behaviours associated with youth violence. Additionally, a self-administered questionnaire will also be used to collect socio-demographic data and data about knowledge, perceptions and behaviours. Study participants will be involved with the principal investigator to develop those tools in interactive group discussions. Prior to involving participants in developing data collection tools, the academic research team will draft the data collection tools based on the existing literature evidence to be modified later on in discussions with participants.

### Implementation fidelity

In the present study, the fidelity components; quality of the delivery of the intervention and participant responsiveness [69] will be considered and the intervention-specific fidelity measures will be developed in an iterative process with the study participants [70]. Mainly the ‘self-report’ method which collects data directly from participants through scales, checklists, etc. which is less time-consuming compared to other methods [71] will be used in the present study. Possible criteria to assess participant responsiveness will be participants’ apparent enthusiasm, level of participation, comprehension of the information, application of the skills and integration of the interventions into life [70, 72, 73]. Possible criteria to assess the quality of delivery of the intervention will be active listening to participants, allowing participants to identify their solutions, encouraging participants throughout the session, asking open questions and facilitating the session, managing time in the session, and tailoring the content as per the needs/suggestions of the participants [70, 74]. The principal investigator will improve the understanding of the student research group about the fidelity criteria. Thereafter, the student research group will decide on the criteria and the response options. Those will be reviewed for clarity [74] with the participants before the implementation.

### Plan for data collection

Before the health promotion intervention, FGDs will be conducted among purposively selected study participants (a subgroup of students) and KIIs will be conducted with key informants like the Principal and teachers of the technical college until the data saturation in the native language; Sinhala by the principal investigator. The students will be selected for FGDs based on the criteria such as gender, seniority, type of studies, etc. At the end of the intervention, again the FGDs and KIIs will be conducted with the same participants to explore the changes in knowledge, perceptions, behaviours and addressed determinants of youth violence. The student research group will be trained on note-taking during the FGDs.

The questionnaire will be used with the students who take part in the study before and after the health promotion intervention. In addition, the principal investigator will maintain a reflective diary and data will be collected through reflective diary notes, field notes, observations, meeting minutes and students’ records throughout the study. Further, fidelity measures will be used to get information about the delivery of the intervention and participant responsiveness. That data will be used to improve the quality of the intervention.

### Plan for data analysis

The FGDs and KIIs will be audio-recorded and transcribed verbatim. The transcribed data will be analysed using the thematic analysis method. The thematic analysis provides detailed, rich but complex data [75] and it identifies common threads or links in data across an interview or set of interviews [76].

Braun and Clarke, 2006 [75] six-step framework thematic analysis method will be used in the present study. As per its steps, after being familiarized with the data, initial codes will be generated. Then, the themes will be searched, reviewed, defined and named. Finally, the report will be produced [75]. In the present study, the data analysis will be a manual process. The academic research team consists of four members will involve in the analysis. Transcribed verbatim will be read line by line and codes will be noted inductively. The common codes that emerged will be identified and agreed upon by them in a group discussion after independent coding. Themes and sub-themes will be identified accordingly.

The list of codes will be discussed with the student research group to generate themes and sub-themes. The analysis done with the academic research team will be compared with these and final themes will be agreed. This will increase rigour in data analysis and help develop the understanding of student research group about the key findings which may later help them to design actions. Data analysis will be done using the original transcripts which are in the native language; Sinhala and only the needed information like themes and quotes will be translated later to English. Data collected from questionnaires will be analysed using descriptive statistics.

Throughout the health promotion intervention, students will continuously reflect and monitor where they are and what the current changes are with the principal investigator. Then, depending on the current progress, the actions can either be modified or new actions can be planned. Thus, this is a rigourous iterative process of data analysis throughout the study. Further, reflective diary notes, field notes, meeting minutes, students’ records and the data from fidelity measures also will be analysed when necessary to triangulate. Ultimately, the overall changes and the experience will be discussed and shared in a forum with the study participants.

Table 2 gives a summary of the plan for data collection, analysis, intended purposes and supported research objectives under the phases; pre-intervention, intervention and post-intervention.

**Table 2:** Plan for data collection, analysis, intended purposes and supported research objectives

Phase of the study	Methods of data collection	Target group	Number of participants	Plan for data analysis	Purpose	Supported research specific objective
Pre-intervention	FGDs	Subgroup of students	Until data saturation	Braun and Clarke, 2006 [75] thematic analysis	To describe the existing knowledge, perceptions and behaviours associated with youth violence	01
	KIIs	Key informants like the Principal and teachers				
	Self-administered questionnaire	All study participants	Approximately 80 students	Descriptive statistics	To quantify the level of knowledge perceptions and behaviours associated with youth violence To collect socio-demographic information	
Health Promotion Intervention	Ongoing data collection through Minutes of group discussions relevant to steps of the health promotion intervention (described in Table 1) Observations Reflective diary notes (mainly of the principal investigator) Field-notes Fidelity measures Any other students’ records	All study participants and principal investigator	Approximately 80 students + principal investigator	Rigourous iterative data analysis throughout the intervention; Quantitative data-descriptive statistics and Qualitative data-thematic analysis	To identify determinants of youth violence To design and implement interventions based on data To monitor periodic changes To modify actions or to plan new actions To reflect and go back in the logical framework (Fig. 1) where necessary To improve the overall quality of the intervention including delivery and participant responsiveness	02, 03
Post-intervention	FGDs	Subgroup of students	Until data saturation	Braun and Clarke, 2006 [75] thematic analysis	To describe the changes that happened in knowledge, perceptions, behaviours and addressed determinants of violence following the intervention	04
	KIIs	Key informants like the Principal and teachers				
	Self-administered questionnaire	All study participants	Approximately 80 students	Descriptive statistics	To quantify the changes that happened in knowledge, perceptions, behaviours and addressed determinants of violence following the intervention	

In addition to during the health promotion intervention, the principal investigator will maintain field notes, a reflective diary and do observations of student behaviours throughout the study including in pre and post-intervention phases. That data also will be considered analysing if necessary

### Study timeline

Before commencing the study with the participants in the real setting, the academic research team required another six months for proposal development through face-to-face meetings, online discussions and email communications. This was an iterative process of reflection that helped the academic research team to develop the methodology and timeline for the present study. That timeline is given in Table 3.

**Table 3:** Study timeline

Years		Year 1						Year 2						Year 3					
Months		2	4	6	8	10	12	14	16	18	20	22	24	26	28	30	32	34	36
Literature review		**×**	**×**	**×**	**×**	**×**	**×**	**×**	**×**	**×**	**×**	**×**	**×**	**×**	**×**	**×**	**×**	**×**	**×**
Phase I: Pre-intervention	Planning and developing data collection tools	**×**																	
	Data collection		**×**																
	Data analysis			**×**	**×**	**×**													
Phase II: Intervention	Step 01				** × **														
	Step 02					** × **													
	Step 03						** × **												
	Step 04							**×**	**×**	**×**									
	Step 05							**×**	**×**	**×**									
Phase III: Post-Intervention	Data collection										** × **								
	Data analysis											**×**	**×**	**×**					
	Writing the report			**×**	**×**	**×**	**×**	**×**	**×**	**×**	**×**	**×**	**×**	**×**	**×**	**×**	**×**	**×**	**×**

*The expected time periods might slightly change when the study is implemented in the real setting

Even though the whole study period is three years, the period of interaction with study participants will be only the first two years. The study period is split into three phases. Phase I is the pre-intervention phase which is the period before the health promotion intervention. It will take around ten months and the engagement of study participants in the research process, planning of the study and setting objectives with them, development of the data collection tools, collection of baseline data and baseline data analysis will be done in this phase.

Phase II will be the intervention phase. There, the health promotion intervention (the steps of the intervention are given in Fig. 1) will be conducted with study participants while the baseline data analysis is going on and will expand to the mid of year 2. The early familiarity of the principal investigator with study participants since phase I will help to engage them in the health promotion intervention and it may reduce the time for step 01 of the intervention. Meanwhile, the analysis of baseline data would be finished and the findings will be used to design and implement locally relevant activities in later steps of the intervention.

Phase III is the post-intervention phase which is the period after the health promotion intervention. It will occupy the rest of the study period. Basically, the post-data collection and analysis will be done to describe the changes that happened following the intervention. Study participants will involve up to a considerable extent in data analysis with the time available for them. Further, this phase includes a session to share experience, learnings and findings.

Additionally, the literature review will be done throughout the study period mainly by the principal investigator. It will support writing the report which may commence four months after the onset of the study.

### Ensuring trustworthiness

Several measures will be taken in the present study to ensure trustworthiness. In the present study, student research group will involve in the data analysis and key findings will be checked with them to increase credibility. Further, qualitative data collection methods which are more appropriate for a PAR [63] will be prominently used. Data will be collected using multiple methods and will be triangulated. Additionally, data will be collected from key informants other than from students till the saturation. All these measures will increase the credibility of the study [77–79]. ‘Prolonged engagement’ or developing an early familiarity with the study participants before the first data collection helps to build up a relationship of trust between the researcher and the participants that increase credibility [78]. Thus, in the present study, participants will work closely with the principal investigator from the stage of developing the data collection tools, before the first data collection.

In the present study, sociodemographic data of the participants will be collected through the questionnaire to provide a clear picture of the setting and the participants which will increase the transferability. The process will be documented and all the study procedures will be justified to facilitate readers to get a clear picture of the decisions made. A reflective diary will be maintained throughout the study period. These measures will ensure the trustworthiness of the study.

### Ethical considerations

The ethical approval for the present study was obtained from the Ethics Review Committee of the Faculty of Applied Sciences, Rajarata University of Sri Lanka (Ref. no. ERC/04/21). Even though there is a mutual relationship between the researcher and the participants in PAR, it is important to have an ethical framework [80]. Participation in this study will be entirely a voluntary decision of the potential study participants and they are allowed to withdraw from the study at any point, up to one month after the study. The college Principal or the academic staff will not involve in the recruitment process. Participants will be informed about the study through an information sheet. Two separate information sheets and consent forms will be used with students and key informants because key informants will participate only in KIIs and two group discussions, not for the whole process. What happens in a PAR cannot be decided accurately at the beginning, thus giving consent to a PAR is like consenting to changes not known already [81]. In the present study, participants will be informed about the possibility of changing the process and their ability to have control over the changes that may happen.

Informed written consent to participate in the study will be taken from the potential participants by the principal investigator after giving sufficient explanations for any questions they have about the study. Participants will be given adequate time to decide their participation and their decision to take part or not to take part in the study will not affect them or their studies. Participation in the FGDs also will be a voluntary decision by the study participants and their written consent for FGDs will be taken in a separate consent form. Study participants will be approached to get their consent after obtaining written permission from the relevant authorities and the Principal of the college on a feasible date and time for them.

Engaging students in a PAR on a sensitive topic like youth violence has the potential to cause retraumatization among victims and it may badly affect the safety of the participants. Thus, our academic research team consists of a psychiatrist and a person with a counselling background who has experience in dealing with sensitive topics and any participant can be referred to them for additional support, whenever needed. It will not be easy to protect the anonymity in PAR within the groups during the research process as the participants will involve and engage with each other in planned activities [82]. However, when reporting the findings, anonymity will be maintained. Each person who takes part in a FGD, KII and fills a questionnaire will be given a participant number, so that their personal details remain confidential. The student research group has access only to anonymized data and confidential information will not be shared.

The time for the study will be allocated in discussion with the administration of the technical college. The principal investigator will get time slots in their academic timetable without disturbing other academic activities. As the present study is planned to be implemented during the COVID-19 pandemic, higher attention will be paid to ethics during the pandemics in addition to the general ethical principles. In pandemics and emergencies, principles such as safety and do-no-harm are critical [83]. In the present study, the safety of the study participants and the principal investigator will be considered a priority and all possible safety precautions such as maintaining physical distance, wearing masks, washing hands, keeping rooms ventilated and avoiding crowds [84] will be followed when engaging with study participants.

## Discussion

The search for effective solutions to youth violence remains a challenge and it is an urgent need to resolve the problem of youth violence systematically with the use of research-based approaches [85]. The strengths, challenges encountered and limitations of the present study are explained below.

### Strengths

PAR has been widely used in other health disciplines such as nursing and mental health [41], but the use of PAR in health promotion is relatively unacknowledged and understated. However, PAR is an important research approach for health promotion [41]. The use of PAR along with health promotion provides a strong theoretical foundation as the key characteristics of health promotion such as empowerment and participation are reflected in PAR [48, 51, 55]. PAR helps to create evidence in areas that lack empirical evidence and create practical solutions [86, 87]. Youth violence in Sri Lankan technical colleges is an area that lacks empirical evidence. Thus, the use of the PAR approach along with a health promotion intervention will be a novel experience for researchers and study participants and it will help to add new knowledge to the existing literature. This research approach itself will actively involve study participants throughout the process. Thus, the study participants may receive a number of benefits from the study. For example, the health promotion intervention will empower them to take actions to reduce youth violence in their college and later it may reduce their risk to become an offender or a victim of future violence. Moreover, students may be able to address youth violence in settings of their everyday life after the study and taking part in the study may build up their competencies and working closely with the principal investigator may develop their research skills that they can apply to do other participatory research as well.

The present study will contribute to generating effective, culturally appropriate, locally relevant youth-led actions that work best for youth in the context of technical colleges or similar settings to address youth violence. Further, the study will provide an evidence base for what works, how and why it works in contrast to the actions decided and led by the professionals. The iterative, spiral nature of PAR [56] and the flexibility of the community-centred health promotion model [52] will help to unfold what works and why it works. The collection of data from diverse methods and the use of the principle of triangulation will also support doing so. Further, the steps that will be taken to ensure the trustworthiness described above also can be identified as a strength. This study will support educational institutes and training colleges where young people are enrolled especially in Sri Lanka and similar LMICs to create supportive and safer environments.

### Challenges encountered and plans to overcome

Three key challenges were encountered during the development of the methodology. The first being certain ethical concerns such as maintaining the anonymity and confidentiality of participants. The plan to overcome that challenge is, to give participant numbers, anonymize all identifiable information and give access only to anonymized transcripts for student research group and not allow them to match individual names with their responses.

Secondly, both health promotion and PAR require an active participant involvement in which participants need to spend a substantial amount of their time in the process. To overcome this challenge, study participants will be guided to integrate the interventions into their routine learning activities and everyday lifestyles. Thirdly, the interventions and possible outcomes to be assessed cannot be predetermined. This will hinder the use of the same data collection tools developed in the pre-intervention phase, in the post-intervention phase as well because some changes that happened following the interventions will not be captured. The plan to overcome that challenge is to have flexibility in modifying the data collection tools in discussion with study participants and triangulating the findings from different sources. Furthermore, the academic research team recognized the importance of keeping records throughout the process to facilitate learning. A reflective diary and a field notebook will be maintained by the principal investigator while the study participants will be guided to keep records of observations and meetings. Those will be considered analysis when recording reflections and triangulating the findings where needed.

### Limitations

There are several limitations to the present study. The present study is planned to be implemented in a technical college where the selection of the study setting and the participants is purposive which may reduce the transferability of the study findings. However, the aim of the study is not to work with a representative sample of students in Sri Lankan technical colleges but to empower youth in a selected technical college to take actions by themselves to reduce violence. And, ultimately to generate effective youth-led actions to address youth violence and to report the procedure followed, changes that happened and lessons learned along with researcher reflections. A larger study of the proposed research can be justified for interested researchers or institutions. Depending on the enthusiasm of the study participants, the occasional closure of the college due to the COVID-19 pandemic and the time available for the principal investigator to interact with students without making changes in their study hours, the present study might take more time than expected.

## Data Availability

Not applicable.

## Abbreviations

FGD: Focus group discussion
GSHS: Global School-based Student Health Survey
HIC: High Income Country
KII: Key informant interview
LMIC: Low and middle income country
PAR: Participatory action research
